# MST1 knockdown inhibits osteoarthritis progression through Parkin‐mediated mitophagy and Nrf2/NF‐κB signalling pathway

**DOI:** 10.1111/jcmm.18476

**Published:** 2024-06-06

**Authors:** Hantao Ye, Tingwen Cai, Yang Shen, Lin Zhao, Haojie Zhang, Jianxin Yang, Feida Li, Jiaoxiang Chen, Xiaolong Shui

**Affiliations:** ^1^ Department of Orthopaedics The Second Affiliated Hospital and Yuying Children's Hospital of Wenzhou Medical University Wenzhou China; ^2^ Key Laboratory of Orthopaedics of Zhejiang Province Wenzhou China; ^3^ The Second School of Medicine Wenzhou Medical University Wenzhou China; ^4^ The Second Affiliated Hospital of Zhejiang Chinese Medical University Hangzhou China

**Keywords:** apoptosis, mitophagy, MST1, osteoarthritis

## Abstract

Osteoarthritis (OA) is a complicated disease that involves apoptosis and mitophagy. MST1 is a pro‐apoptotic factor. Hence, decreasing its expression plays an anti‐apoptotic effect. This study aims to investigate the protective effect of MST1 inhibition on OA and the underlying processes. Immunofluorescence (IF) was used to detect MST1 expression in cartilage tissue. Western Blot, ELISA and IF were used to analyse the expression of inflammation, extracellular matrix (ECM) degradation, apoptosis and mitophagy‐associated proteins. MST1 expression in chondrocytes was inhibited using siRNA and shRNA in vitro and in vivo. Haematoxylin–Eosin, Safranin O‐Fast Green and alcian blue staining were used to evaluate the therapeutic effect of inhibiting MST1. This study discovered that the expression of MST1 was higher in OA patients. Inhibition of MST1 reduced inflammation, ECM degradation and apoptosis and enhanced mitophagy in vitro. MST1 inhibition slows OA progression in vivo. Inhibiting MST1 suppressed apoptosis, inflammation and ECM degradation via promoting Parkin‐mediated mitophagy and the Nrf2‐NF‐κB axis. The results suggest that MST1 is a possible therapeutic target for the treatment of osteoarthritis as its inhibition delays the progression of OA through the Nrf2‐NF‐κB axis and mitophagy.

## 1 INTRODUCTION

Osteoarthritis (OA) is chronic articular disease that is characterized by the degradation of cartilage and the emergence of secondary osteophytes. OA gradually affects the joints' cartilage or the entire joint, including the joint capsule, subchondral bone, surrounding muscles and synovial membrane.^1^

A growing body of research has identified inflammation as a factor in the early progression of OA.^2^ It causes the body to produce a large number of inflammatory cytokines.^3^ These cytokines promote the release of enzymes responsible for making cartilage matrix, causing a shift in the metabolism of the extracellular matrix (ECM) from producing matrix to breaking it down. This transition is characterized by the production of MMPs (matrix metalloprotein) and ADAMTS (A Disintegrin and Metalloproteinase with Thrombospondin motifs) enzymes, rapidly breaking up articular cartilage.^4^, ^5^, ^6^

Multiple studies have demonstrated that the dysregulation of autophagy and apoptosis in chondrocytes is crucial in advancing OA.^7^, ^8^, ^9^ Mitophagy is a specific type of autophagy. Autophagy selectively degrades damaged or defective mitochondria, eliminating them before they threaten cell viability.^10^ Ensuring mitochondrial homeostasis is of utmost importance. Furthermore, the occurrence of the disease is influenced by mitochondrial dysfunction. In recent years, an increasing number of studies have demonstrated that mitochondrial damage plays a role in OA,^11^ rheumatoid arthritis (RA),^12^ intervertebral disk degeneration (IVDD) and posttraumatic osteoarthritis (PTOA).^13^, ^14^

Nuclear factor erythroid 2‐related factor 2 (Nrf2) is a transcription factor that participates in several physiological activities and maintains cellular homeostasis.^15^ The activation of Nrf2 plays an anti‐inflammatory and anti‐oxidative role.^16^ In addition, studies have shown that the NF‐κB (nuclear factor κ‐B, p65) pathway regulates inflammation and the catabolism of cartilage extracellular matrix.^17^, ^18^, ^19^ When IL‐1β stimulates, the phosphate group is characterized for proteasomal degradation and connected to NF‐κB inhibitor α (IκBα). Consequently, it facilitates the relocation of p65 to the nucleus, stimulating the inflammatory response and leading to the degradation of the extracellular matrix.^20^, ^21^ Multiple studies have demonstrated that the activation of Nrf2 inhibits the NF‐κB signalling pathway, leading to a decrease in the breakdown of the extracellular matrix and exhibiting an anti‐inflammatory effect.^22^, ^23^, ^24^ As a result, the Nrf2/NF‐κB axis is considered an essential target for the management of OA.

MST1 (mammalian sterile 20‐like kinase 1) is a core component of the Hippo signalling pathway.^25^ By regulating apoptosis progression and cell proliferation, this signalling pathway maintains a dynamic balance between tissue environment and organ size. MST1 is a pro‐apoptotic factor^26^ that could promote apoptosis by phosphorylating the forkhead box O (FOXO) transcription factor.^25^ According to reports, MST1 contributes to developing mitochondrial dysfunction and apoptosis in rheumatoid arthritis.^25^ MST1 has been shown to decrease mitophagy in ageing‐related diseases.^27^ In addition, it significantly contributes to the development and progression of diseases such as diabetic cardiomyopathy,^28^ tumours,^29^ and mental disorders.^30^ Several studies have demonstrated that MST1 promotes cartilage degradation in OA.^31^, ^32^

However, the mechanism by which MST1 controls the process of chondrocyte degeneration remains uncertain. At present, no definitive study investigates the potential correlation between chondrocyte apoptosis, autophagy, or other related aspects. The treatment of OA with drugs targeting MST1 lacks a solid theoretical base. Therefore, using a series of in vitro and in vivo analyses, this study aims to investigate the precise mechanism of MST1 regulating the progression of OA to provide a theoretical base for the future development of targeted therapeutics.

## 2 MATERIALS AND METHODS

### 2.1 Ethical Approval

Compliance with all regulations was strictly enforced during all surgical procedures and animal aftercare. With the requirements of the Wenzhou Medical University Animal Care and Use Committee, human cartilage tissue was obtained from the Second Hospital of Wenzhou Medical University, and experiments on human cartilage tissue were approved by the Ethics Committee of the Second Hospital of Wenzhou Medical University (ethic code: LCKY2022‐K‐267‐01), and the experiments were carried out in accordance with the guidelines of Helsinki Declaration.

#### 2.1.1 Reagents and chemicals

3‐Methyladenine (3‐MA) (C6H7N5; HPLC ≥99%; Cat# M9281) and tert‐butyl hydro‐peroxide (TBHP, (CH3)3COOH; Cat# 416665) solution were provided by Sigma‐Aldrich (St. Louis, MO, America). Safranin‐O (Cat# G1371), dimethyl sulfoxide (DMSO, C2H6SO, Cat# 67‐68‐5) and Collagenase II (Cat# 9001‐12‐1) were purchased from Solarbio (Beijing, China). Aggrecan and Collagen II antibodies, MMP13 (matrix metallopeptidase 13), p62 (Sequestosome‐1, SQSTM1), Bcl‐2 (B‐cell lymphoma‐2) and Bax (BCL2‐associated X protein) antibodies were purchased from Abcam (Cambridge, MA, United States; Cat#ab3778, Cat#ab34712, Cat#ab219620, Cat#ab109012, Cat#ab18285); Cell Signalling Technology (Danvers, MA, United States; Cat#4211, Cat#9661, Cat#12741) provided antibodies against Parkin, Cleaved‐Caspase 3, p65, p‐p65 and LC3 (Microtubule‐associated protein 1 light chain 3, MAP1LC3). Antibodies against β‐Actin, iNOS (inducible nitric oxide synthase), COX‐2 (Cyclooxygenase‐2) were purchased from Proteintech (Chicago, IL, United States). The secondary antibodies labelled with Alexa Fluor 488 and Alexa Fluor 594, respectively, were obtained from Abcam (Cambridge, MA, United States; Cat#ab150073, Cat#ab150108). 4,6‐Diamidino‐2‐phenylindole (DAPI) was obtained from Yaseen Biochemical (Shanghai, China), while the reagents for cartilage culture were obtained from Gibco (Grand Island, NY, United States).

#### 2.1.2 Collection and evaluation of human cartilage specimens and chondrocytes

Cartilage samples were obtained from six patients with OA who underwent total knee arthroplasty (age 50–67; that is, 58.6 years of age; Kellgren–Lawrence classification grade III or IV). Six healthy cartilage specimens from trauma patients without OA and without distinguishing clinical and radiological features (age 51 to 65 years; Kellgren‐ Lawrence grades 0 or I). A portion of cartilage tissue was removed for histological analysis. The minced cartilage tissue was washed three times in phosphate‐buffered saline (PBS) and then digested with collagenase II (2 mg/mL) at 37°C for 4 h. The samples were then centrifuged, washed three times with PBS, resuspended in Dulbecco's Modified Eagle Medium: F‐12 (DMEM/F12) and incubated at 37°C, 5% CO_2_.After washing the chondrocytes twice with PBS, the original medium was replaced every other day with a fresh medium.

#### 2.1.3 siRNA transfection

The si‐MST1 and si‐Nrf2 were purchased from OBiO Technology (Shanghai). Inoculated cells were cultured in six‐well plates for 3 h to achieve a 60%–70% cell density. This was followed by the addition of 50 nM of control or siRNA duplexes transfection cells, which were achieved using Lipofectamine 3000 (Thermo Fisher, UT, USA). The cells were subjected to further treatment before being used for Western Blot assays.

#### 2.1.4 Western Blot (WB)

Chondrocytes were treated accordingly to extract proteins and then incubated with corresponding antibodies at 4°C. After one night, the cells were incubated with HRP‐conjugated secondary antibody for 1 h at room temperature. Finally, the protein bands were measured under the imaging system, and the images were processed using Image J.

#### 2.1.5 Cellular mitochondrial isolation

We purchased Cell Mitochondria Isolation Kit from Beyotime Biotechnology. The cells were washed once with PBS, digested with Trypsin‐ethylenediaminetetraacetic (EDTA) Solution and then centrifuged at 100 *g* for 5 min at room temperature. The cells were gently re‐suspended in PBS that had been pre‐chilled in an ice bath. After removing a small portion to count, the remaining cells were centrifuged at 600 *g* for 5 min at 4°C to precipitate. The mitochondrial isolation reagent should be added, the cells should be gently suspended, cooled for 10 min in an ice bath, homogenized for 10–30 strokes and the cell suspension centrifuged at 600 *g* for 10 min at 4°C.

#### 2.1.6 Alcian blue staining

Alcian Blue Stain Kit was purchased from Solarbio (Beijing, China).For paraffin sections, dewax to distilled water. Then, soak in Alcian acidic solution for 3 min. And stain with Alcian staining solution for 30 min. Washing in running tap water for 1 min. Dehydrate in series of alcohol, transparent by xylene and seal with resinene.

#### 2.1.7 Immunofluorescence assay

After corresponding treatment, the chondrocytes were washed three times with PBS, fixed with 4% paraformaldehyde at room temperature for 15 min and blocked with 5% goat serum for 1 h. Antibody incubations were then performed overnight at 4°C. Then the cells were washed, incubated with goat anti‐rabbit or goat anti‐mouse secondary antibody for 1 h at room temperature, finally stained with DAPI, and mounted.

#### 2.1.8 Animal model

Regarding the choice of sample size, we refer to previous studies.^33^ The experimental animals were 25 10‐week‐old C57BL/6 strain wild‐type (WT) male mice purchased from the Wenzhou Medical University's Experimental Animal Center (licence No. SCXK [ZJ] 2015–0001), Zhejiang province, China. Destabilisation of the medial meniscus (DMM) was used to develop a mouse model of OA., as previously described.^34^ To anaesthetize the mice, 40 mg/kg of pentobarbital was intraperitoneally administered. After opening the joint capsule using microsurgical shears and cutting open the meniscus and medial meniscal ligament, the joint capsule was re‐cut medially to the patellar tendon. Similarly, sham‐operated mice were also opened the joint capsule without cutting the medial meniscus ligament. We injected 10 μL of AAV (Adeno‐associated virus) into the joint cavity via the transpatellar tendon approach on days 0, 15, 30 and 45 after DMM. Mice The mice were randomly divided into five groups: Sham, DMM, Sham+sh‐NC, DMM + sh‐NC and DMM + sh‐MST1, with 5 mice in each group. We anaesthetized the mice 8 weeks postoperatively, sacrificed them with pentobarbital, and examined their knee joints dissected and histological.ly.

#### 2.1.9 Histopathologic analysis

We embedded the processed joint tissues in microscope slides and stained them with Safranin O‐fast green (SO) to measure their cytoarchitecture and morphology and subchondral bone. Mouse knee tissues were scored using the Osteoarthritis Research Society International (OARSI) score.^35^ Each mouse group had five histological scores. The specific grouping of the mice was known only to Xiaolong Shui, and the operator conducting the experiments remained blind to the grouping.

#### 2.1.10 Immunohistochemical analysis

Sections were dewaxed, hydrated, and antigen retrieved, then blocked with 5% goat serum for 1 h and incubated with primary antibodies overnight at 4°C. Wash three times with PBS and incubate with HRP‐conjugated secondary antibody at 37°C for 2 h.

#### 2.1.11 Flow cytometry

Chondrocytes from mice were harvested, and the apoptosis levels were assessed using an Annexin V‐FITC/PI apoptosis kit (MultiSciences Biotech, China). After labelling, samples were examined using a Beckman flow cytometer (Beckman Coulter, USA).

#### 2.1.12 Statistical analysis

Based on independent experiments, means and standard deviations (SDs) are shown. The normality of the parameters was assessed by the Kolmogorov–Smirnov test, and student's *t*‐test and one‐way analysis of variance (ANOVA) with Tukey's post hoc test were used to analyse the parameters of the normal distribution. The Kruskal–Wallis H test was used to analyse the histological score, which did not follow a normal distribution. *p* < 0.05 was statistically significant, and analysis was done using GraphPad Prism 9 (La Jolla, CA, USA).

## 3 RESULTS

### 3.1 MST1 is upregulated in osteoarthritic human and DMM mice

**FIGURE 1:**
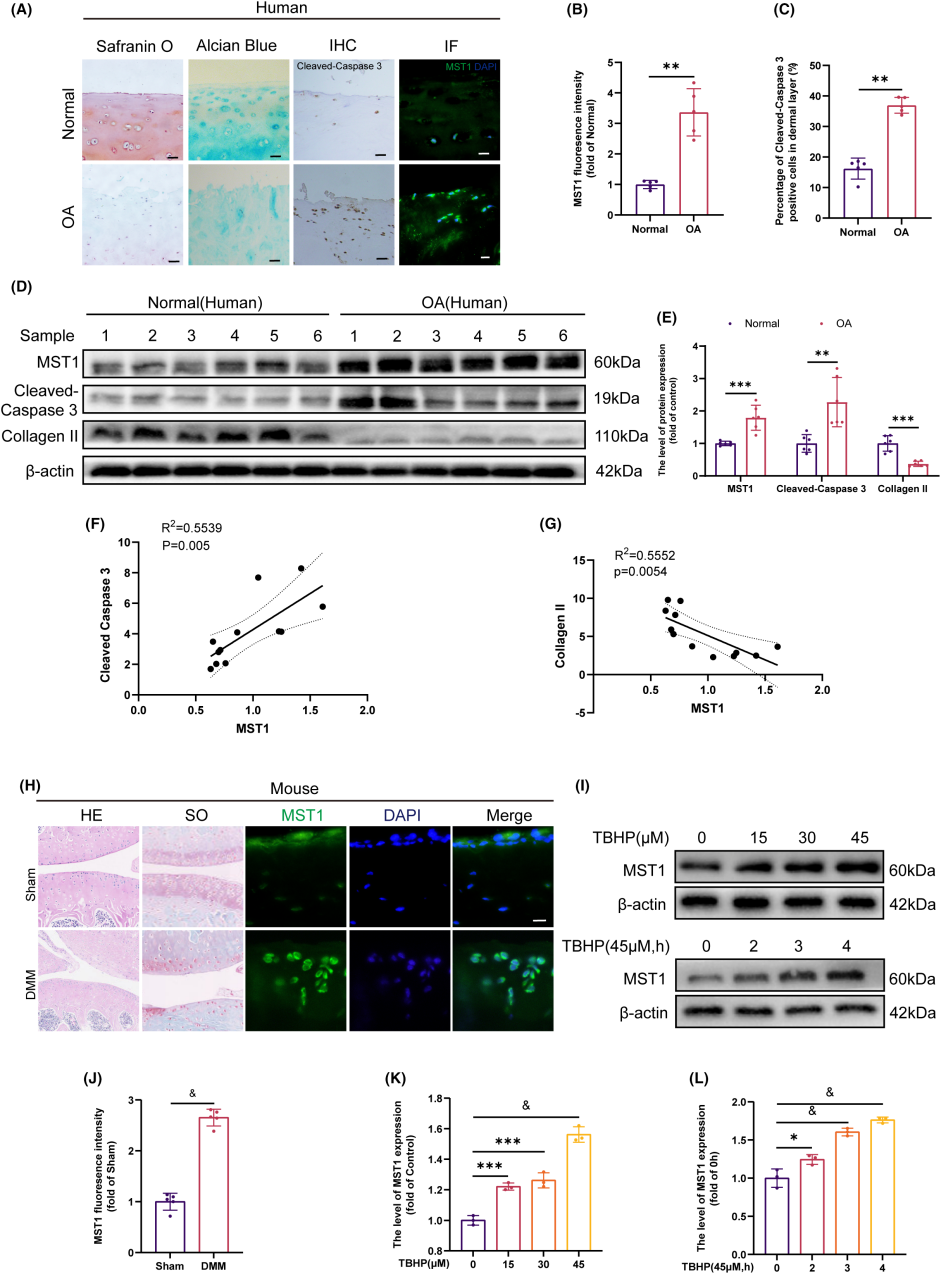
MST1 was upregulated in osteoarthritic human and DMM mice. (A, B, C) Results of Safranin O staining, Alcian Blue staining, immunohistochemistry staining and immunofluorescence staining of human articular cartilage from healthy people and patients with OA (Scale bar: 50 μm, *n* = 5). (D, E) Protein levels of MST1 in human chondrocytes derived from healthy individuals and OA patients (*n* = 6). (F, G) Linear correlation analysis between MST1 and Cleaved‐Caspase 3, MST1 and Collagen II. (H) Results of HE staining and SO staining of mice articular cartilage (Scale bar: 50 μm, *n* = 5). (H, J) Immunofluorescence images showing MST1 expression (Scale bar: 50 μm, *n* = 5). (I, K, L) The expression levels of MST1were measured by WB in chondrocytes (*n* = 3). Data are presented as the mean ± SD. According to statistical analysis, the differences between groups were significant: **p < 0.01, ***p < 0.001, ^&^ *p* < 0.0001.

To explore the relationship between the underlying mechanisms of OA and MST1, the expression of MST1 in the cartilage tissue of healthy individuals and OA patients was analysed. These results revealed significant Cleaved‐Caspase 3 expression in OA‐affected tissues, demonstrating a high level of apoptosis in OA patients' cartilage tissues **(**Figure 1A,C **)**. The Alcian blue staining and Safranin O staining were used to stain cartilage tissue. The findings indicated that cartilage tissue degradation was more prominent in patients with OA **(**Figure 1A **)**. The IF findings reveal that the fluorescence intensity of MST1 is higher in OA tissue than in normal tissue **(**Figure 1A,B **)**. The WB of chondrocytes in individuals without health issues and those with OA (OA) revealed elevated levels of MST1 and Cleaved‐Caspase 3 expression. In comparison, Collagen II expression was reduced in OA patients. A correlation study was carried out on MST1, Collagen II and Cleaved‐Caspase 3. MST1 has a strong inverse association with Collagen II and a large positive correlation with Cleaved‐Caspase 3, (Figure 1F,G). As mentioned above, the findings suggested that the levels of MST1 were elevated in OA tissues, which was strongly associated with the breakdown of the ECM and apoptosis. A DMM model was developed to provide further support for the increased expression of MST1 during the pathological development of OA. The occurrence of cartilage breakdown in DMM, as shown by the HE and SO stains displayed in Figure 1H, demonstrated the effective development of the mouse model of OA. The IF staining results agreed with the findings in OA tissues (Figure 1H,J). Furthermore, the chondrocytes were isolated from mice and subjected to TBHP stimulation to replicate in vitro OA chondrocytes, following a prior protocol.^36^ The results showed that MST1 exhibited a concentration and time increase dependent on the extending duration and increasing concentration of TBHP stimulation (Figure 1I,K,L).

### 3.2 Knockdown of MST1 attenuates apoptosis, inflammatory response and ECM degradation

**FIGURE 2:**
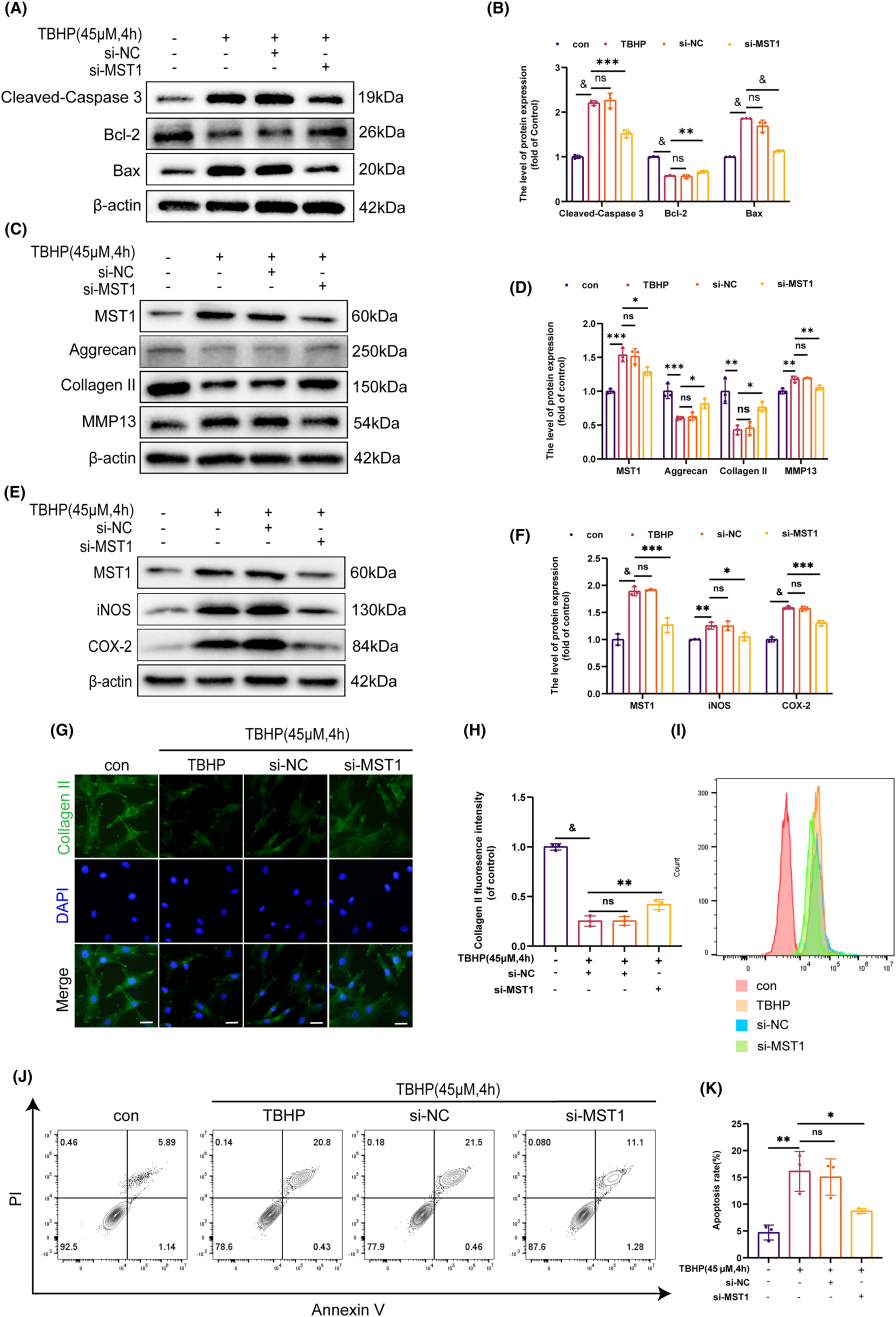
Knockdown of MST1 attenuates apoptosis, inflammatory response and extracellular matrix degradation. (A, B) The expression levels of Cleaved‐Caspase 3, Bax and Bcl‐2 were measured by WB in chondrocytes. (C, D) The expression levels of MST1, Aggerecan, Collagen II and MMP13 were measured by WB in chondrocytes. (E, F) The expression levels of MST1, iNOS and COX‐2 were measured by WB in chondrocytes. (G, H) Immunofluorescence images showing Collagen II expression. (I) Detection of ROS levels in chondrocytes by flow cytometry. (J, K) The TBHP‐treated chondrocytes apoptosis rates were counted with flow cytometry and analysed with Image J. Data are presented as mean ± SD (*n* = 3); *p < 0.05, **p < 0.01, ***p < 0.001, ^&^ *p* < 0.0001.

The present study used siRNA to transfect chondrocytes and decrease MST1 expression to study the effect of MST1 on the programmed cell death of mouse chondrocytes. Cleaved‐Caspase 3 and Bax expression decreased after transfection, whereas Bcl‐2 expression increased **(**Figure 2A,B **)**. Furthermore, the si‐MST1 group demonstrated higher levels of Aggrecan and Collagen II than the TBHP group, but MMP13 expression was lowered **(**Figure 2C,D **)**. The degree of inflammation is a crucial element that impacts the advancement of OA.^2^ Therefore, the WB was used to measure the levels of protein expression. In contrast to the TBHP group, the si‐MST1 group had lower levels of iNOS and COX‐2 expression, demonstrated in Figure 2E,F. Using IF staining, the degree of ECM breakdown was also evaluated. Figure 2G,H shows that the fluorescence intensity of Collagen II in si‐MST1 was much higher than in the TBHP group, adding to the evidence that supports the earlier WB results. Increased ROS generation causes mitochondrial damage and promotes OA progression.^37^ Hence, the ROS level in chondrocytes was assessed using flow cytometry **(**Figure 2I **)**. Interestingly, the ROS level was significantly lower in the si‐MST1 group than in the TBHP group. In addition, the rate of apoptosis in chondrocytes after different treatments was measured using flow cytometry. The si‐MST1 group significantly reduced the apoptosis rate (Figure 2J,K). Together, these results suggest that MST1 inhibition reduces chondrocyte apoptosis.

### 3.3 Knockdown of MST1 activates nuclear translocation of Nrf2 and inhibits NF‐κB signalling pathway

**FIGURE 3:**
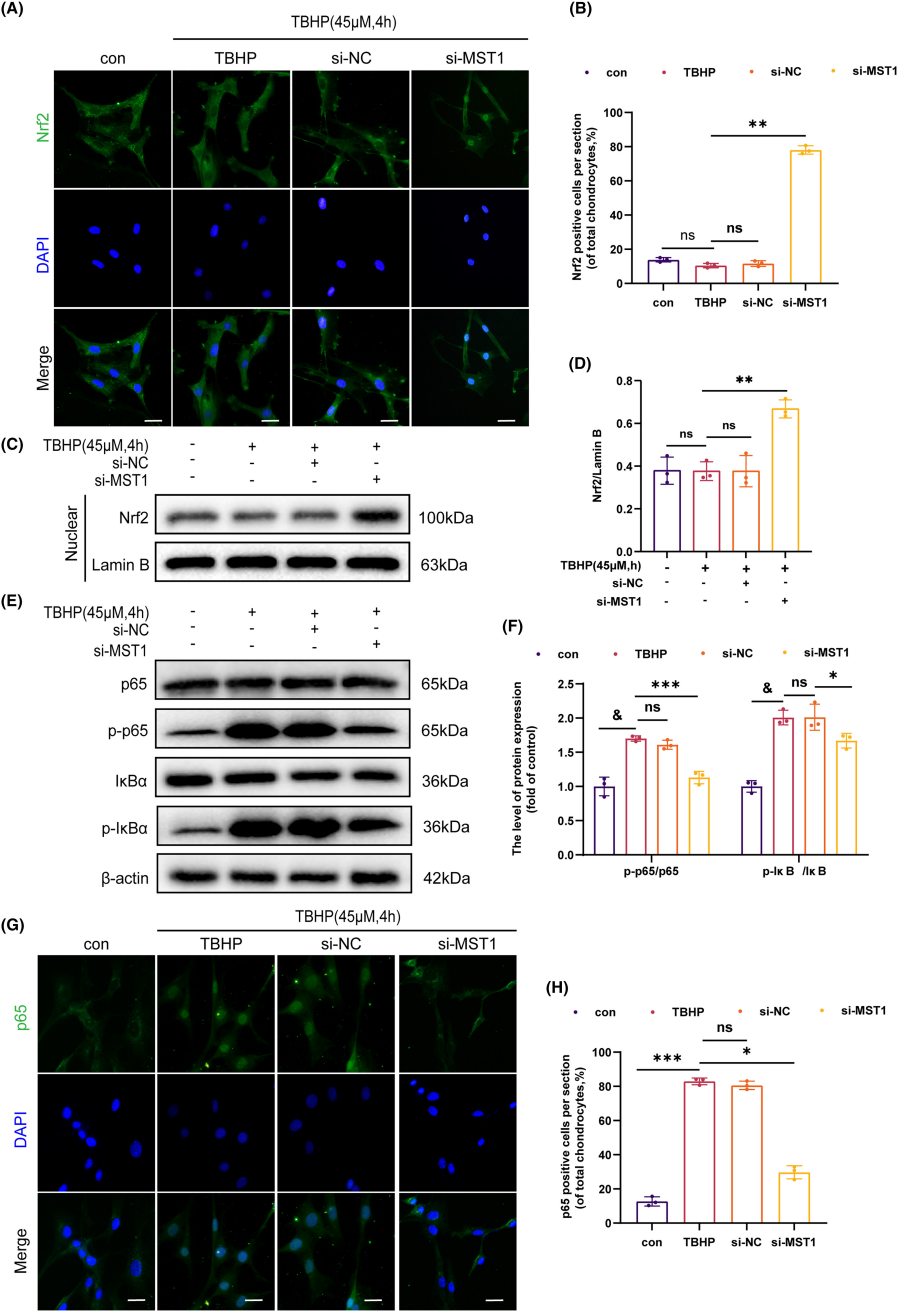
Knockdown of MST1 activated nuclear translocation of Nrf2 and inhibits NF‐κB signalling pathway (A, B) Immunofluorescence images showing Nrf2 expression (B, D) The level of Nrf2 expression in chondrocyte nuclear. (E, F) The expression levels of p65, p‐p65, IκBα and p‐IκBα were measured by WB in chondrocytes. (G, H) Immunofluorescence images showing p65 expression. Date are presented as the mean ± SD (*n* = 3); *p < 0.05, **p < 0.01, ***p < 0.001, ^&^ *p* < 0.0001.

The activation of Nrf2 controls inflammation and levels of ROS in chondrocytes. Moreover, inflammation and extracellular matrix breakdown are significantly influenced by the NF‐κB signalling pathway. Thus, it was hypothesized that MST1 controls the Nrf2 and NF‐κB signalling pathways to govern the inflammatory response and ECM degradation in chondrocytes. The levels of Nrf2 expression were determined using IF staining. The expression of Nrf2 in the nucleus increased significantly in the si‐MST1 group **(**Figure 3A,B **)**. Afterwards, the WB was used to determine the Nrf2 protein expression level in the nucleus. These results showed that the si‐MST1 group had higher levels of Nrf2 expression in the nucleus **(**Figure 3C,D **)**. Moreover, the WB and IF labeling was utilized to determine how inhibiting MST1 affected the NF‐κB signalling pathway **(**Figure 3E–H **)**. When comparing the si‐MST1 group to the TBHP group, there was a significant reduction in the nucleus expression of p65. The results indicate that blocking MST1 activates Nrf2 while inhibiting the NF‐κB signalling pathway from activating.

### 3.4 Inhibition of MST1 inhibits the NF‐κB signalling pathway by activating Nrf2 to reduce inflammation and ECM degradation

**FIGURE 4:**
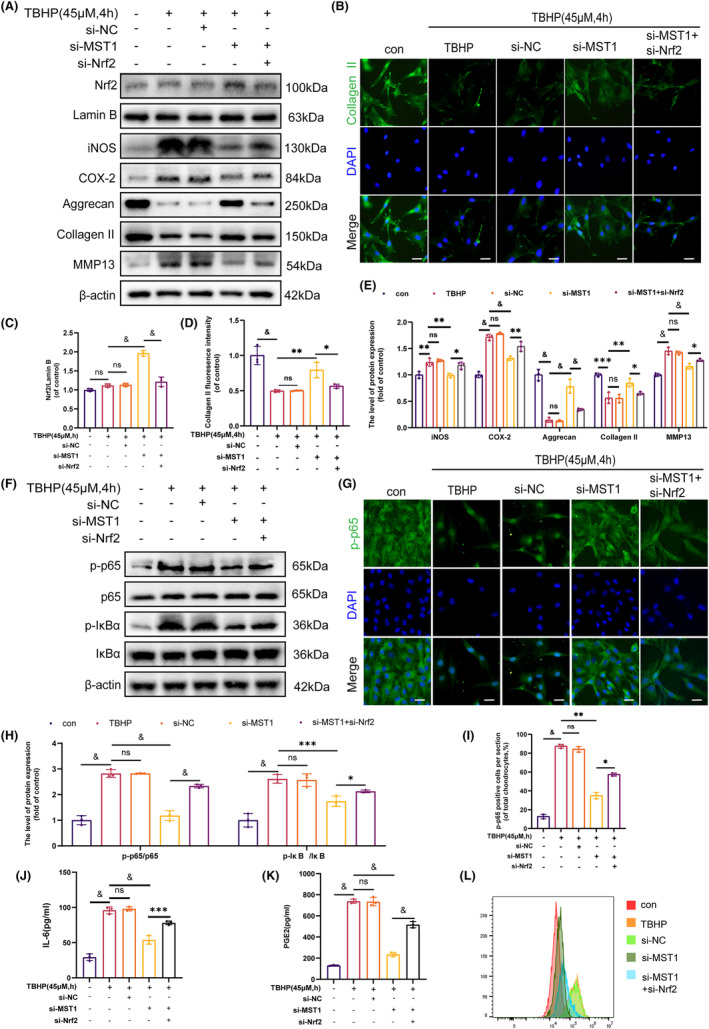
Inhibition of MST1 inhibited NF‐κB signalling pathway by activating Nrf2 to reduce inflammation and ECM degradation. (A, C, E) The protein level of Nrf2, iNOS, COX‐2, Aggrecan, Collagen II, MMP13 were assessed by WB. (B, D) Immunofluorescence staining of Collagen II in chondrocytes (Scale bar: 50 μm). (F, H) The protein expression of p65, p‐p65, IκBα and p‐IκBα in mice chondrocytes determined by WB. (G, I) Immunofluorescence staining of p65 in chondrocytes (Scale bar: 50 μm). (J, K) ELISA detection of the levels of IL‐6 and PEG2 in the cell culture supernatant after the specified treatment. (L) Detection of ROS levels in chondrocytes by flow cytometry. Data are expressed as the mean ± SD (*n* = 3); *p < 0.05, **p < 0.01, ***p < 0.001, ^&^ *p* < 0.0001.

To investigate the effect of MST1 on the NF‐κB pathway, the Nrf2 expression was inhibited using siRNA **(**Figure 4A,C **)**. The WB revealed that the inhibition of Nrf2 led to increased expression levels of p‐p65 and p‐IκBα **(**Figure 4F,H **)**. Furthermore, the IF staining of p65 yielded results similar to those of the WB **(**Figure 4G,I **)**. Moreover, following the reduction of Nrf2 expression, as shown in Figure 4A–E, the levels of iNOS, COX‐2 and MMP13 were elevated, whereas Collagen II and Aggrecan levels were lowered. Similarly, the results of the ELISA ascertained a rise in the expression levels of PGE2 and IL‐6 after the suppression of Nrf2 expression **(**Figure 4J,K **)**. The flow cytometry results of ROS also indicated an increase in ROS levels following the inhibition of Nrf2 **(**Figure 4L **)**. These results suggest that MST1 expression suppression inhibits the NF‐κB signalling pathway by activating Nrf2, which lowers inflammation and ECM breakdown in chondrocytes.

### 3.5 Inhibition of MST1 expression promotes Parkin expression and activates mitophagy

**FIGURE 5:**
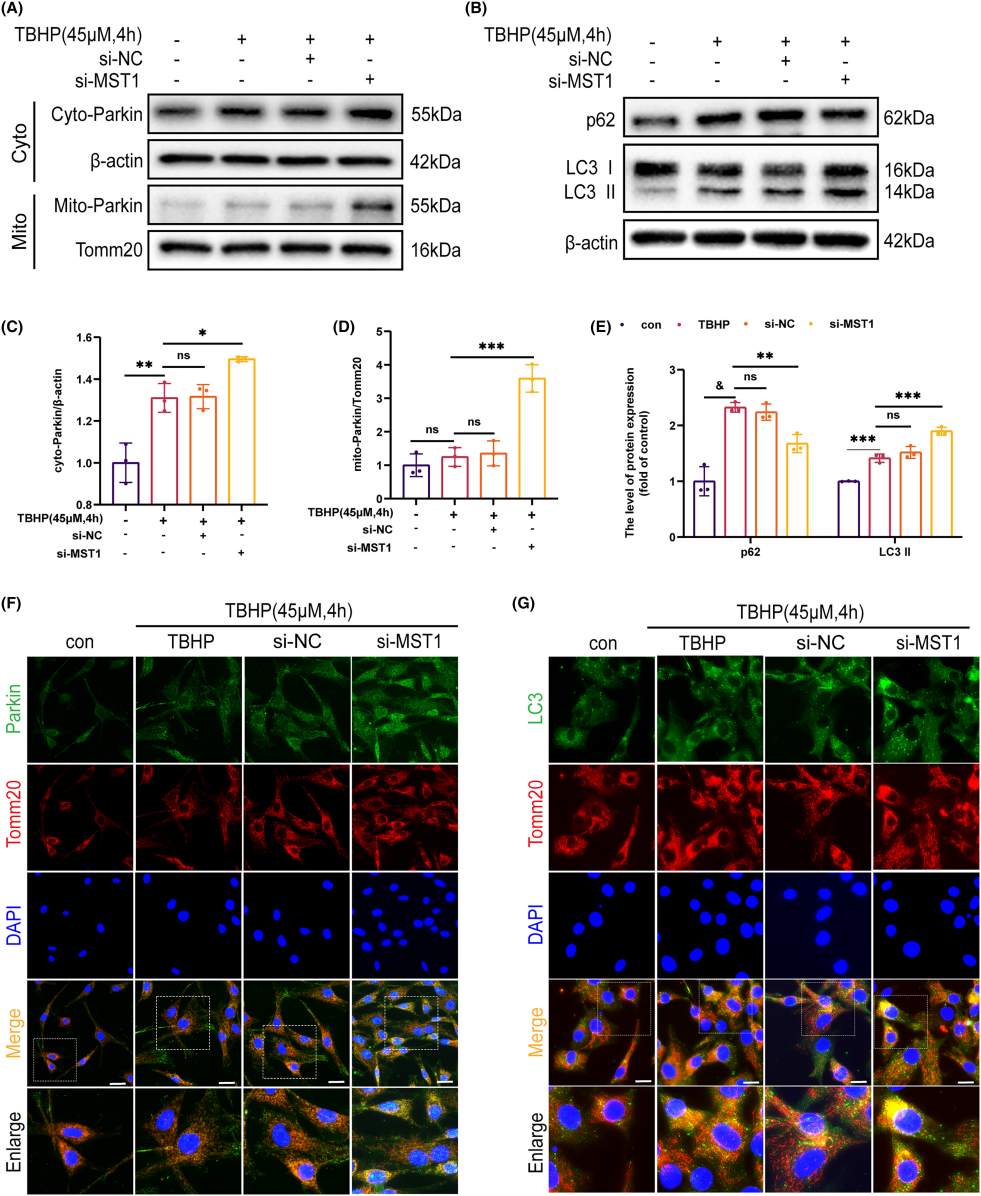
Inhibition of MST1 expression promoted Parkin expression and activates mitophagy. Mice chondrocytes were pre‐exposed to si‐MST1 and then treated with TBHP (45 μM, 4 h). (A, C, D) The WB assay showing the expression levels of Parkin in mitochondria and cytosol presented as a ratio. (B, E) p62 and LC3 II/I levels after treatment with TBHP or si‐MST1. Mice chondrocytes were treated according to the treatment regimens described above. (F) Immunofluorescence staining of Parkin and Tomm20 in chondrocytes treated as described above (Scale bar: 10 μm). (G) Immunofluorescence double staining of LC3 and Tomm20 on chondrocytes treated as described above (scale bar:20 μm). Data are presented as the mean ± SD (*n* = 3); *p < 0.05, **p < 0.01, ***p < 0.001, ^&^ *p* < 0.0001.

Studies have indicated that mitophagy is a protective mechanism when OA is present. Therefore, to determine if MST1 promotes apoptosis through mitophagy, the relationship between MST1 and mitophagy was studied. Compared to the pre‐depletion condition, Figure 5A,C,D show that the depletion of MST1 increased Parkin manifestation, specifically on mitochondria (mito‐Parkin). However, TBHP stimulation alone did not increase mito‐Parkin. The IF staining was used to designate mitochondria and co‐localise Parkin using the mitochondrial outer membrane indicator Tomm20. These findings indicate that the binding fluorescence intensity of Parkin was notably elevated in the knockdown group **(**Figure 5F **)**. Figure 5B,E shows increased levels of P62 and LC3 II in chondrocytes treated with TBHP as observed in the WB, indicating that autophagic flux was blocked. When MST1 was inhibited, p62 levels significantly decreased and LC3 II expression increased **(**Figure 5B,E **)**, restoring autophagic flow. The findings suggest that mitophagy is facilitated by inhibiting MST1. Mitophagy clears dysfunctional mitochondria. Hence, the autophagic vesicles were labelled with LC3 and Tomm20. Figure 5G shows that in the knockdown group, co‐localization of LC3‐positive autophagosomes and mitochondria was more common than in the non‐knockdown group, suggesting that MST1 suppression promotes mitophagy. In summary, Parkin's translocation to mitochondria is facilitated by inhibiting MST1 expression, which can improve mitophagy.

### 3.6 Inhibition of MST1 expression inhibits apoptosis by promoting Parkin‐mediated mitophagy

**FIGURE 6:**
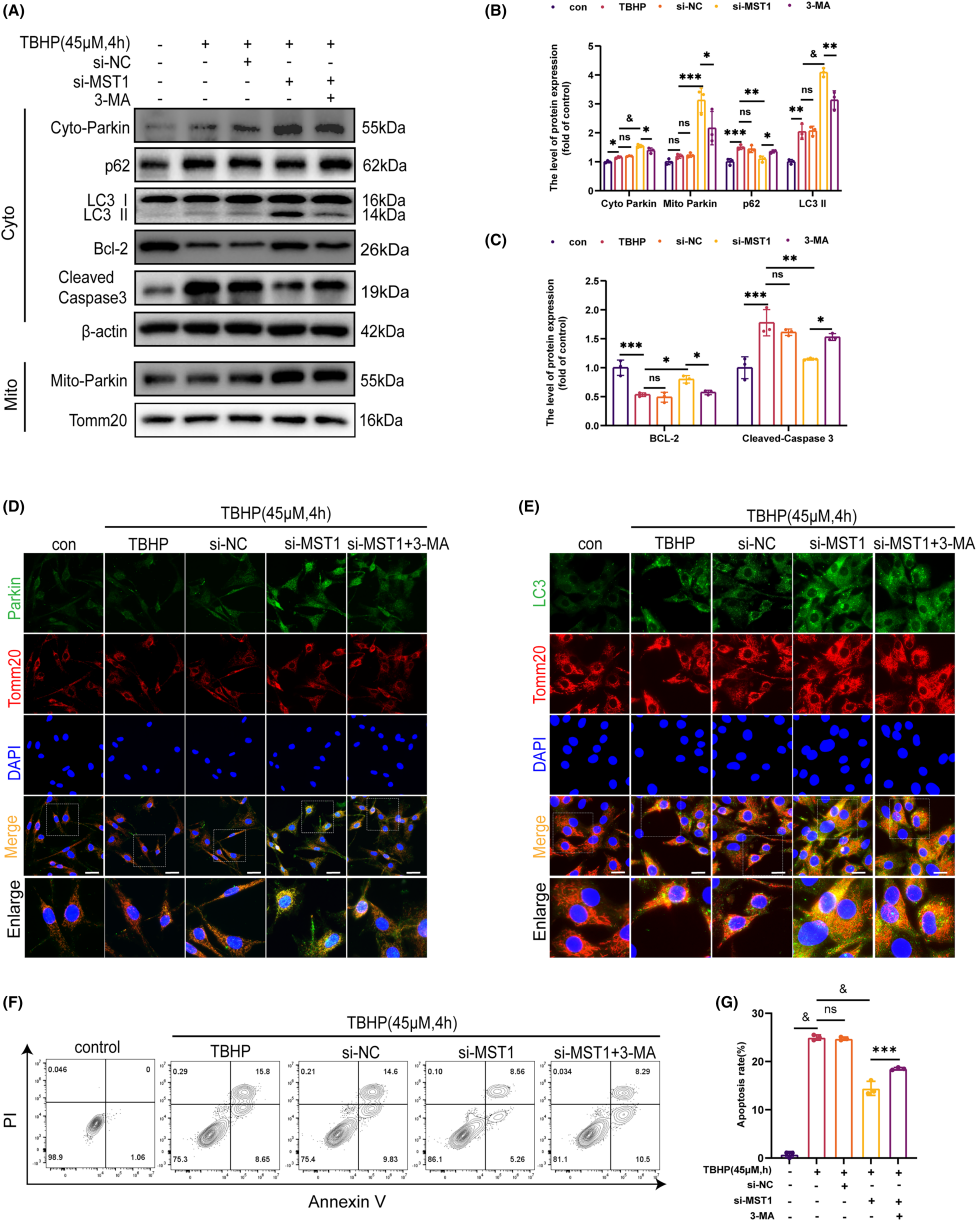
3‐MA reversed the protective effect of inhibiting MST1 expression on chondrocytes. Mice's chondrocytes were first exposed to si‐MST1 and 3‐MA (10 mM for 2 h) and then treated with TBHP (45 μM for 4 h). (A, B, C) Results of WB assay showing that Bcl‐2, Cleaved‐Caspase 3, p62, LC3 and Parkin expression levels were significantly elevated in chondrocytes treated as detailed above. (D) Immunofluorescence double staining of Parkin and Tomm20 in chondrocytes treated as described above (Scale bar: 50 μm). (E) Immunofluorescence staining of LC3 and Tomm20 in chondrocytes treated as described above (Scale bar: 50 μm). (F, G) The chondrocytes apoptosis rates were counted with flow cytometry and analysed with Image J. Data are presented as the mean ± SD (*n* = 3); *p < 0.05, **p < 0.01, ***p < 0.001, ^&^ *p* < 0.0001.

The findings mentioned above indicate that suppressing MST1 expression enhances mitophagy. To investigate the role of MST1 in apoptosis regulation through Parkin‐mediated mitophagy, the autophagy inhibitor 3‐Methyladenine (3‐MA) was employed to suppress mitophagy. The WB experiment demonstrated that after MST1 knockdown, LC3 II, cytoplasmic Parkin and mitochondrial Parkin expression levels increased, whereas p62 expression levels decreased **(**Figure 6A,B **)**. However, 3‐MA reversed these effects. Furthermore, the mitochondrial outer membrane marker Tomm20 was used to co‐localise Parkin after performing IF staining of the mitochondria. It was found that the knockdown group had significantly higher Parkin binding fluorescence intensity than the 3‐MA group **(**Figure 6D **)**. Moreover, the LC3 and Tomm20 co‐localization was carried out using IF staining. The findings demonstrated that the binding fluorescence intensity of LC3 and Tomm20 increased following MST1 knockdown. However, 3‐MA reversed these effects **(**Figure 6E **)**. Furthermore, WB indicated that the MST1 knockdown led to a decrease in the expression level of Cleaved‐Caspase 3 and increased in the expression level of Bcl‐2.

In contrast, an opposite effect was observed in the 3‐MA group **(**Figure 6A, C **)**. In addition, the chondrocyte apoptosis rate was counted using flow cytometry, as shown in Figure 6F,G. The rate of chondrocyte apoptosis was higher in the 3‐MA group than in the MST1 knockdown group, thereby validating the prior results. In summary, suppressing the production of MST1 triggers the process of Parkin‐mediated mitophagy, which in turn prevents apoptosis.

### 3.7 MST1 knockdown ameliorated OA in mice

**FIGURE 7:**
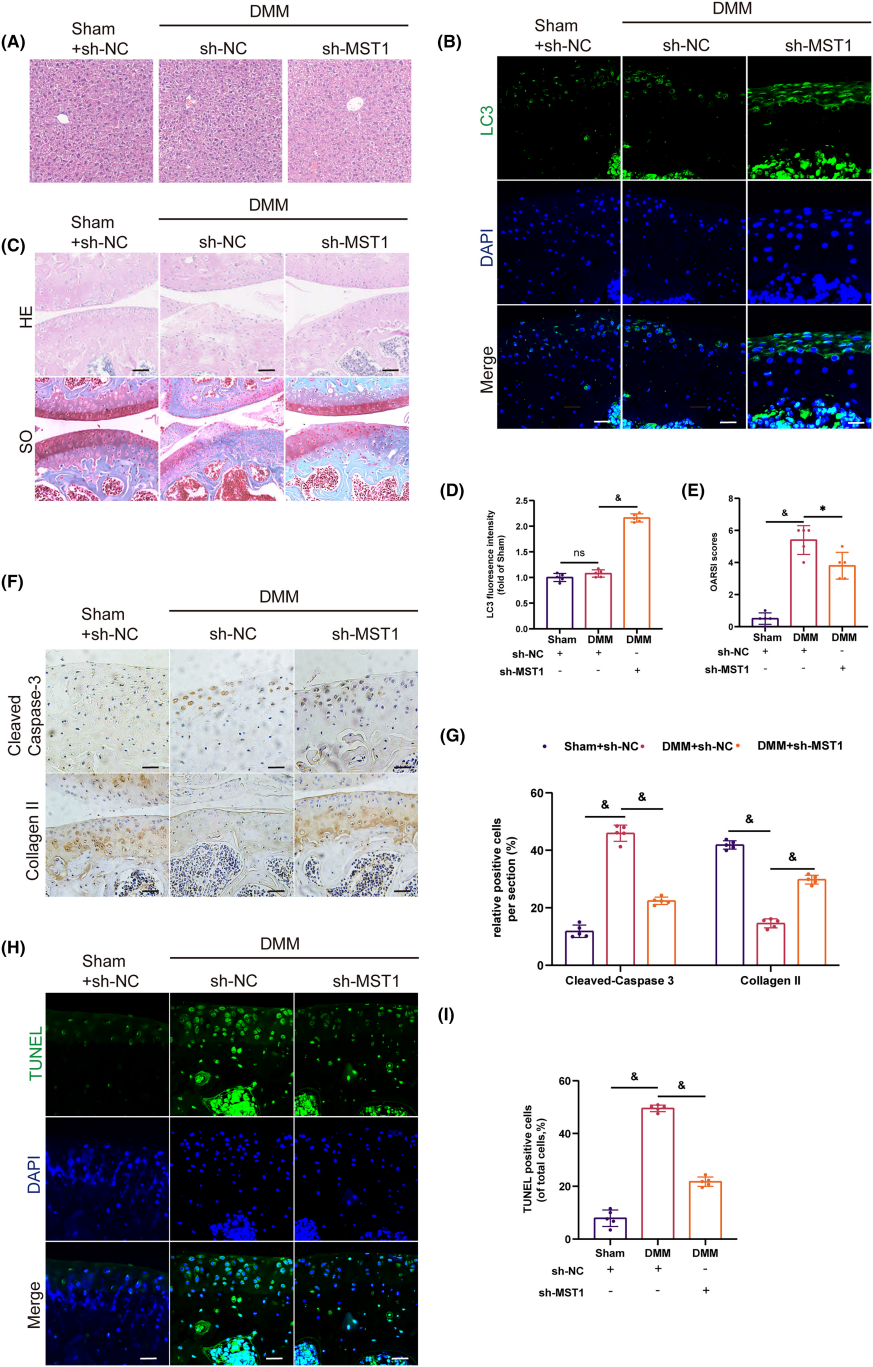
MST1 knockdown reversed the progression of OA in vivo. (A) H&E staining of mice at 8‐week‐post‐surgery. (B, D) Immunofluorescence of LC3 8 weeks after surgery (Scale bar, 50 μm). (C) Results of HE staining and SO staining of mice articular cartilage. (E) The scores of cartilages based on OARIS. (F, G) Immunohistochemistry of Cleaved‐Caspase 3 and Collagen II at 8‐week‐post‐surgery (Scale bar: 50 μm). (H, I) Mice cartilage staining with TUNEL. (Scale bar: 50 μm). Data are presented as the mean ± SD (*n* = 5); *p < 0.05, **p < 0.01, ***p < 0.001, ^&^ *p* < 0.0001.

Further research was carried out to examine the in vivo function of MST1. The mouse OA model was created through the destabilisation of the DMM surgery. Throughout the 8 weeks following the surgery, the present study employed H&E and SO staining techniques to evaluate any possible histomorphological differences. To determine the effect of suppressing MST1 on the liver, the H&E staining was conducted on mice liver samples, asMST1 is mainly synthesized in the liver. This allowed us to examine any histological changes. The results revealed no apparent variations in mice's livers before and after MST1 inhibition **(**Figure 7A **)**. In the knockdown group, LC3 IF labelling revealed increased mitophagy after MST1 inhibition **(**Figure 7B,D **)**. Then, a histomorphology analysis was carried out using Safranin O staining. According to the study, 2 months after surgery, the articular cartilage surface in the DMM + sh‐NC group was degraded, which resulted in a lower cell count than in the knockdown group **(**Figure 7C **)**. Furthermore, the DMM + sh‐MST1 group exhibited a significantly reduced OARSI score compared to the DMM + sh‐NC group **(**Figure 7E **)**. The results of the immunohistochemical staining showed that the sh‐MST1 group had higher expression of Collagen II and less expression of Cleaved‐Caspase 3 **(**Figure 7F,G **)**. Furthermore, the tissue sections were stained using TUNEL. Compared to the DMM + sh‐NC group, a significant decrease was observed in positive cells in the knockdown group **(**Figure 7H,I **)**, confirming reduced chondrocyte apoptosis levels following MST1 knockdown. In summary, inhibition of MST1 expression lowers apoptosis levels by encouraging mitophagy, which slows the progression of OA in mice.

## 4 DISCUSSION

Despite the high prevalence of OA, the intricate nature of its pathophysiology has led to a limited availability of effective treatments. In OA, the mechanisms of inflammation, programmed cell death (apoptosis) and degradation of ECM play a vital role in the deterioration of cartilage.^38^, ^39^, ^40^ MST1 acts as a pro‐apoptotic factor in the Hippo signalling pathway. Multiple research studies have demonstrated that inhibiting MST1 has a suppressive effect on inflammation in cells.^41^, ^42^ This study showed a substantial level of cellular apoptosis in chondrocytes inside tissues obtained from patients with OA. An increased expression of MST1 in these specific tissues was found for the first time.

Furthermore, blocking MST1 reduced Bax, Cleaved‐Caspase 3, MMP13, iNOS and COX‐2 levels while simultaneously increasing the Bcl‐2, Collagen II and Aggrecan levels. These data suggest that decreasing MST1 may have the ability to reduce chondrocyte death, decrease ECM disintegration and lessen inflammation. However, it should be noted that this hypothesis has not been previously demonstrated or reported. When the expression of both Nrf2 and MST1 was reduced simultaneously, it was observed that there was an increase in both inflammation and ECM degradation. This suggests that inflammation and ECM degradation were reduced because of the activation of Nrf2 when MST1 was blocked. Meanwhile, the activation of the NF‐κB signalling pathway was detected. These findings indicate that the inhibition of MST1 expression leads to suppressing the activity of the NF‐κB signalling pathway through the activation of Nrf2.

The OA's development is strongly connected to the preservation of chondrocyte balance. Eliminating damaged and malfunctioning mitochondria and preserving chondrocyte stability are two crucial roles of mitophagy.^43^, ^44^ Several signalling mechanisms regulate mitophagy. Among the most well‐known signalling pathways is the PINK1‐Parkin pathway. Mitophagy regulation is strongly affected by its involvement.^45^, ^46^

The development of autophagic vesicles requires the conversion of soluble LC3 (LC3I) into the variant linked with autophagic vesicles (LC3II). Hence, the LC3II/LC3I ratio functions as a crucial marker for autophagy.^47^ During autophagy, certain autophagy‐related proteins, such as p62, are incorporated into fully developed autophagosomes and then undergo degradation.^48^ This study observed that the levels of mitophagy in chondrocytes were elevated following TBHP treatment compared to those in the control group. Elevating mitophagy levels could be a compensating reaction by chondrocytes to safeguard their well‐being. Therefore, the levels of Parkin, P62 and LC3 II/LC3 I were also elevated, similar to previous studies by others.^13^, ^49^

However, as there was no significant rise in the levels of mito‐Parkin/cyto‐Parkin within the mitochondria, it indicated that MST1 impeded the transfer of Parkin from the cytoplasm to the mitochondria and disrupted the process of autophagy. This was further validated by the differences detected in the escalating p62 level concentration and the extent of mitophagy. In comparison, inhibiting MST1 resulted in to a notable increase in mito‐Parkin/cyto‐Parkin, a decrease in p62 expression relative to the initial condition and an increase in LC3 II/LC3 I, indicating an enhanced level of mitophagy and the restoration of autophagic flux. A group in a follow‐up study treated with 3‐MA, an autophagy inhibitor, was added to support these findings further. This research found that chondrocyte apoptosis decreased and mitophagy increased upon inhibition of MST1. After mitophagy was inhibited, the therapeutic benefit of MST1 inhibition was reversed. It was hypothesized that suppressing MST1 expression may prevent apoptosis by inducing mitophagy.

There are several limitations to this study. At first, it was unclear if MST1 and Parkin interacted directly or if MST1 regulated Parkin through multiple pathways. Secondly, there is a possibility that more signalling pathways related to OA have not been investigated. Further research is required to determine the role of MST1 in OA both in vivo and in vitro.

## 5 CONCLUSION

The findings suggest inhibiting MST1 may reduce apoptosis by enhancing Parkin mitochondrial translocation and mitophagy. Similarly, MST1 suppresses the inflammatory response in osteoarthritis. It decreases the breakdown of the ECM by promoting the movement of Nrf2 into the cell nucleus, thereby weakening the activity of the NF‐κB signalling pathway. MST1 is, thus, suggested for the first time as a possible target for treating OA.

## AUTHOR CONTRIBUTIONS

**Hantao Ye:** Data curation (equal); investigation (equal); supervision (equal); validation (equal). **Tingwen Cai:** Data curation (equal); formal analysis (equal); software (equal); writing – original draft (equal). **Yang Shen:** Investigation (equal); validation (equal); writing – original draft (equal). **Lin Zhao:** Formal analysis (equal); methodology (equal); writing – original draft (equal). **Haojie Zhang:** Data curation (equal); supervision (equal); visualization (equal); writing – original draft (equal). **Jianxin Yang:** Methodology (equal); software (equal); validation (equal). **Feida Li:** Data curation (equal); methodology (equal); resources (equal); visualization (equal). **Jiaoxiang Chen:** Conceptualization (equal); project administration (equal); resources (equal); writing – review and editing (equal). **Xiaolong Shui:** Data curation (equal); funding acquisition (supporting); resources (equal); validation (equal).

## FUNDING INFORMATION

This study was supported by funds from the Nature Science Foundation of Zhejiang Province (LGF21H060009) and the Science and Technology Plan Project of Wenzhou Municipality (Y20220207, Y2020051).

## CONFLICT OF INTEREST STATEMENT

Authors declare that they do not have any competing interests.

## Data Availability

Data available on request from the authors.
